# Flood vulnerability in township informal settlements

**DOI:** 10.4102/jamba.v18i1.1833

**Published:** 2026-03-31

**Authors:** Sazi W. Gcabashe, Sareesha Pillay

**Affiliations:** 1 Department of Public Management and Leadership, Faculty of Humanities, Nelson Mandela University, Gqeberha, South Africa

**Keywords:** informal settlements, floods, vulnerability, townships, Nelson Mandela Bay Municipality, integrated vulnerability, urbanisation

## Abstract

Migration has shaped townships into hubs of urban informality that are characterised by the propagation of informal settlements. With their expansion, informal settlements have become increasingly vulnerable to the devastating impacts of hazards. This article explored factors that influence flood vulnerability in township informal settlements in the NMBM. Framed by the Integrated Vulnerability Approach by McEntire, the study collected secondary data sources from reputable online repositories and digital media platforms using keywords. Data were analysed through thematic analysis. The findings reveal that flood vulnerability in the case study is conceived by a combination of the nature and location of informal settlements. The population characteristics of informal settlement dwellers and the marginalisation of infrastructural public service drive them to occupy hazard prone vacant land in ‘stressed areas’. Despite the risk factor of their hazard-prone locations, informal settlement dwellers hold a ‘sense of place’ because of socioeconomic locational advantage and cultural resonance with their settlements that keeps them in their settlements. The study contends that flood vulnerability in informal settlements in townships occurs in combination of biophysical and social vulnerability. The study recognises that the population characteristics of the informal settlements are influenced by migration and its resultant socioeconomic conditions for low-skilled migrants.

**Contribution:**

The study demonstrates that the conception of informal settlements in townships and the legacies thereof are key origins of the infrastructural, public service delivery socioeconomic deficits in these settlements that drive the interactions of biophysical and social vulnerabilities.

## Introduction

Rapid urbanisation is expanding the population and spatial density of South African urban geographies. This trend reflects the broad context of socio-political and spatial dynamics of urban areas that are driven by migration, economic pressures, spatial inequalities and persistent housing backlogs. In urban townships particularly those located in metropolitan municipalities, these factors often cause urban informalities that emerge outside regulated spatial planning and durable housing infrastructure (Morolle et al. 2022; Williams-Bruinders & De Wit 2020). This has led to the mushrooming of informal settlements established on environmentally and structurally vulnerable areas that lack proper infrastructure, planning and municipal services. For instance, informal settlements in urban townships tend to be located on unstable slopes, low-lying floodplains and former landfills, which expose residents to environmental hazards such as floods (Mokhele et al. 2024).

In the Nelson Mandela Bay Municipality (NMBM), flood hazards are a recurring issue in township informal settlements. The persistent threats from these disasters are heightened by their lack of infrastructure, limited access to reliable municipal services and the marginal geographic positioning of their highly and densely populated settlements (De Wit 2011; Lupindo 2015; Williams-Bruinders & De Wit 2015). This risk is compounded by the vulnerability experienced by residents who face systemic socioeconomic challenges that undermine their ability to withstand and recover from the impacts of hazards. Moreover, people tend to adopt disparate and unsustainable strategies of survival that exposes them to hazards that affect their health and environmental well-being (Pelling 2003; Reis 2021).

While the NMBM has progressively employed efforts to improve the precarious biophysical and socioeconomic conditions in informal settlements through service delivery and infrastructural development, informal settlements remain vulnerable to flood hazards. Moreover, floods continue to inflict the loss to property and life of a largely low-skilled and poor migrant population. Researchers have attributed this issue to the failure of local authorities to integrate township informal settlements into the municipality’s integrated disaster risk reduction planning framework (Sibanda 2016). As a result, the municipality continually reacts to the impacts of floods rather than proactively manage their risks and effectively perpetuate long-term risk and vulnerability. The prevalence of meteorological hazards in informal settlements is not unique to the NMBM but exist in a global context. Studies have found that informal settlements have become hazard hotspots recognised ‘… for both their development potential to increase disaster risk and their high level of vulnerability to disasters’ (Johnson et al. 2020 in Reis 2021:1). To put differently, ‘… disaster hazards with existing informal settlement vulnerability and exposure serves as disaster traps to the poor’ (Abunyewah et al. 2017:244).

Reducing the risk of disasters at a local level must be embedded on a comprehensive understanding of vulnerability because understanding vulnerability empowers local authorities to develop risk-informed approaches that consider the unique spatial and socioeconomic context of communities at risk (UNISDR 2015). It is therefore critical to probe the factors that contribute to flood vulnerability within the unique context of township informal settlements in the NMBM. Against this backdrop, this study aims to investigate the nature of flood vulnerability in township informal settlements situated in NMBM. Our study is informed by the McEntire’s model of the Integrated Vulnerability Approach (IVA) (McEntire et al. 2010) and focuses on flood risks.

## Research methods and design

This study is based on an extensive qualitative review of literature on hazard vulnerabilities in township informal settlements using the NMBM as the case study. According to Coombs (2022:1), ‘[*A*] case study is a methodical research approach used to generate an in-depth understanding of a contemporary issue of phenomenon in a bounded system’.

Data were sourced and downloaded from empirical and theoretical studies, official municipal publications and digital media sources. The search was conducted on Google Scholar, Google News, South Eastern Association Libraries (SEALS) and NMBM’s official municipal sites using key terms including: ‘informal settlements in the NMBM’, ‘townships’, ‘township vulnerabilities in the NMBM’, ‘disasters in townships’, ‘flood vulnerability’ and ‘flooding in informal settlements in the NMBM’. These key terms were used to narrow the search for sources on hazards, resilience and coping capacities in relation to the risk of flood vulnerability in township informal settlements within the case study.

The researchers retrieved secondary data sources that documented disasters affecting informal settlements in townships in the municipality. Sources were considered if they specifically mentioned the risk and impacts of flood hazards in informal housing located in townships within the NMBM. For context, there are approximately 55 000 informal households in 156 informal settlements across the municipality (NMBM 2023b). Of these, 146 are located within the townships of Ibhayi (11), Northern Areas (10), Kareiga (5) and Motherwell (3) (NMBM 2023b). The selection of sources was purposely isolated to these areas. However, our interest later extended to urban informalities that are located among formal township settlements. The study refers to these structures as ‘back room accommodation’.

To select the sources for the article, the researchers employed a two stage criteria that included: 1) identify the relevance of the source to the study, and (2) determine the source’s applicability to flood vulnerability in informal settlements in townships located in the NMBM. Articles that passed this preliminary manual review were analysed through thematic analysis to organise the meanings derived from the text. The themes were carefully organised to enable the researchers to synthesise the findings in a coherent manner under the theoretical framework.

## Conceptual and theoretical framework

### Vulnerability

Vulnerability is a broad term that holds various conceptions from different disciplines and fields of study. In its disaster context, the term details the circumstances that increase the susceptibility of people to the impacts of hazards (Wu et al. 2023). These conditions are determined by physical, social, economic and environmental factors, which interact to define the coping capacity of people or a community (Singh et al. 2014). Bamweyana et al. (2020) assert that these factors are drawn from the physical environment and its socioeconomic features, as well as the political dynamics that characterise a particular geography. Within this understanding, emphasis is adequately placed on community and its capacity to prepare for, mitigate, respond and recover from the impacts of hazards.

Socioeconomic and physical environment attributions of vulnerability imply that the term is a confluence of elements; each of which is associated with features that influence the presence and kind of vulnerability that exists in a community. For instance, Delilah Roque et al. (2020) make note of biophysical vulnerability, which refers to a community’s geographic proximity to a hazard. Biophysical vulnerability confirms the presence and proximity of hazards relative to the physical location of a community, and focuses on this physical characterisation in making communities vulnerable to the impacts of hazards. For this reason, it is regarded as a function of exposure and sensitivity. This notion of vulnerability is mainly applied in research that study natural hazards in changing physical environments (see Blöschl 2022). On the other hand, Blaikie et al. (1994) reference social vulnerability, which is concerned with the socioeconomic and demographical factors that determine the degree of susceptibility a community faces to the effects of hazards. In this regard, vulnerability is explored by the implications of properties such as race, gender, education level, employment status, income and strength of social networks. This implies that population characteristics indicate the degree of vulnerability to hazards because they determine the capacities of communities to cope with the effects of hazards. Furthermore, hazards affect communities differently according to their population characteristics where those on the lower end of the socioeconomic spectrum are most susceptible to the impacts of hazards. In support, Flanagan et al. (2011) found that disaster studies have shown that socially vulnerable groups are ‘… more likely to die in a disaster event and less likely to recover after one’. Social vulnerability is thus a function of resilience.

Various conceptions of disaster have highlighted the need to view vulnerability holistically in terms of the preparation, mitigation, response and recovery of the impacts of hazards. This is on the basis that the impacts of hazards are adverse in vulnerable people – both in the biophysical and social sense. In addition, ‘… [*v*]ulnerability factors often occur in combination’ (Morrow 1999 in Flanagan et al. 2011:3). The chosen theoretical framework for this research (discussed below) reflects this holistic perspective.

### Integrated vulnerability approach

The integrated vulnerability approach (IVA) offers a multidisciplinary perspective to frame vulnerability and its effect on hazards in society. Drawing on the tenets of the Pressure and Release Model (or Disaster Crunch Model), the IVA combines features of social vulnerability and physical vulnerability to understand the factors that subject people to the effect of hazards. The theory was conceptualised by McEntire following the recognition that existing theoretical perspectives on disaster could not encapsulate the situations in which disasters emerge from the early 2000s and in the future (Lagadec 2006). The IVA thus factors human characteristics and activities that render communities susceptible to physical events. In this regard, vulnerability is framed according to the effects of social and economic systems as well as what Boin and Hart (2006:46) refer to as ‘innocent factors’, which include socioeconomic status, gender, ethnicity and race among others. The combination of these factors merges forces that expose people, communities and organisations to the effects of hazards and the subsequent occurrence of disasters, suggesting that the factors that drive vulnerability and disasters are linked to the structural (physical) and social dynamics of a society (Buckle 2005).

According to Poteyeva et al. (2006) in McEntire et al. (2010), the multidisciplinary nature of the IVA facilitates a systematic approach that incorporates the contributions of engineering, physical sciences, social sciences and organisational management. These disciplines conceptualise vulnerability differently in relation to preventing and preparing for disasters. For instance, the physical sciences school expresses that the location of human settlements relative to their proximity to hazards exposes areas to threats of disasters (McEntire et al. 2010). Conversely, the engineering school focuses on creating ‘resilience’ through the construction and reinforcement of the built environments. This school contends that the inadequacy of the quantity, design and quality of physical infrastructure determines the level of vulnerability a settlement is subjected to (McEntire et al. 2010). For example, structures that are built with dubious construction materials and the lack of critical infrastructure face increased vulnerability and subsequent heightened exposure to hazards. The structural school, which is based on social ideals, emphasises the effects of socioeconomic and demographic factors (herein after referred to as population characteristics) in leading to hazard vulnerability (McEntire et al. 2010). Lastly, the organisation school focuses on ‘… the effectiveness of response and recovery operations, and stresses how preparedness, leadership and manage determines the ability to adapt [*to*] disasters’ (McEntire et al. 2010:57).

In isolation, these perspectives show clear limitations by failing to conceptualise vulnerability holistically (McEntire 2005). This supports Lewis and Mioch (2005) who argue that a single framing of vulnerability reduces the meaning of the term. Together, however, the variables expand dynamics that determine factors that: push people to settle in high risk areas (physical science school), prohibit the building of safer human settlement structures (engineering school) and consider the location of human settlements (structural school). McEntire’s IVA presents an integration of these schools by drawing on the links between the foundational conceptions of each, namely coping capacity, resilience and exposure (Manandhar & McEntire 2014; McEntire et al. 2010).

This expression of the IVA model provided the researchers a guideline to scrutinise hazard vulnerability in township informal settlements in the NMBM because it ‘… incorporates insights from the physical world emphasizes the roles of social, economic, and political relations in creating hazardous situations’ (Singh et al. 2014:75). To put differently, this approach focuses on ‘[*T*]he characteristics of individuals and their relationship with society in wider context, the nature of their relationships and the physical and societal environment they inhabit’ (Singh et al. 2014:75). In his development of the model, McEntire argued that the integration can be achieved by identifying the complex interactions between the types of variables beyond the structural and social causes of disasters (McEntire et al. 2010).

## Literature review

### The urban geography of South African townships

The term township was conceptualised by the Apartheid regime and used to identify marginalised segregated urban areas reserved for non-whites (Hunter & Posel 2012 in Membele et al. 2021). According to Williams-Bruinders and De Wit (2020), these settlements were located on the outskirts of the urban centres that were reserved for white South Africans. Empowered by the *Group Areas Act of 1948*, the National Party government prohibited land tenure and restricted access to urban centres for non-whites by enforcing Pass laws (colloquially termed *Dompass*) following the economic boom in the 1970s that necessitated the influx of cheap black migrant labour into urban centres (Lemon 2021; Membele et al. 2021; Webb 2023; Williams-Bruinders & De Wit 2020).

According to Barry and Rüther (2005), the apartheid system during this time allowed for the infux of black migrants in informal settlements across urban centre. However, conflicts arose between local and national authorities as this influx contradicted racial segregation. Therefore, cities were pressured by national authorities to provide housing that would conform to the segregatory national laws and policies that maintained the spatial status quo. This was particularly pertinent in cities such as Port Elizabeth that were mostly composed of white and coloured populations and subsequently expect to receive considerable influxes of black migrant labourers. To respond to the pressures, local governments provided small matchbox-type housing on the outskirts of urban towns and cities (Williams-Bruinders & De Wit 2020). Interestingly, the development of these settlements were proposed to alleviate the socioeconomic and health issues that affected *unserviced* urban plots that migrants had initially occupied (DeMoss 2012). Among these were better sanitation and larger properties – aspects that served to entice black migrants into voluntarily moving to these areas. However, as Monama et al. (2022) and Modise (2019) highlights, townships were and remain organised as overpopulated clustered dormitory settlements that are poorly serviced by the government. Consequently, these settlements became spaces for social challenges such as health, poverty and unemployment (Moghayedi et al. 2023). These issues have continued despite the democratic dispensation in 1994, with socio-political and socioeconomic dynamics thrusting further ills such as high crime rates, drug abuse, HIV/AIDS, youth unemployment and the propagation of informal settlements. This is in the wake of townships remaining densely populated squalors that subject people to precarious living conditions.

### Informal settlements in townships as a urbanisation effect

The notion of human settlements is important and serves as a critical variable to the context of townships amid modern-day dynamics of urbanisation that shape the physical and social landscapes of these geographies. Arguably, the most poignant factor is the cheap cost of living in townships and how this attracts migrants and immigrants (Malgas & Zondi 2020; Scheba & Turok 2020). Abrahams and Everratt (2019:256) argue that mass influxes into townships because of its affordability appeal extends capacities of the regulated formal housing market that is often unable to cater for the housing needs of low-skilled and economically deprived segment of work-seekers who constitute the largest number of migrants. In part, formal housing is simply unaffordable for this demographic who are often compelled to seek cheaper and unregulated alternatives. In townships, this comes in the form of informal settlements.

Informal settlements are subject to much debate owing to the interchangeable use of the term with conceptions such as slums, shanty towns, squatter settlements and unplanned towns (Abunyewah et al. 2017:239). Although these interchangeable terms seem to apply to different contexts, their central precept is the illegal occupation of vacant land. Moreover, this land is often outside land-use schemes and without planning permission. Satterthwaite et al. (2020) clarify that informal settlements are located on land that has not been demarcated for human settlements whereby spatial planning and environmental assessments have been conducted to test their suitability and integration into existing infrastructure and socioeconomic systems. As a result, they lack basic infrastructure and municipal services such as water and sanitation, stormwater infrastructure and electricity reticulation among others (Nyashanu et al. 2020; Wiltgen et al. 2021).

The literature notes that informal settlements globally are frequently located close to biophysical hazards such as industrial zones and flood plains that, while posing health and safety threats, offer employment opportunities, informal business activities and access to social resources (Williams et al. 2019). For instance, Satterthwaite et al. (2020) postulate that the illegal occupation of land for shack dwellings is driven by economic motives whereby people want to be located in an area that is close to employment opportunities and social infrastructure. Furthermore, informal settlements are dense, clustered and overcrowded settlements that consist of unstable makeshift houses built with unconventional building materials. These characteristics render these settlements vulnerable to hazards. Accordingly, low-income communities experience vulnerability that encompass geographic, socio-economic and attitudinal factors.

The establishment of new informal settlements, expansion of existing freestanding informal settlements and proliferation of informal backyard housing structures in townships indicate that informal settlements have spiralled beyond municipal control. For instance, informal households have grown from 1 170 902 in 1995 to 1 294 904 in 2011 and 1 300 000 in 2016 (Nkonki-Mandleni et al cited in Membele et al. 2021). According to Jeffery (2010), the increase in the number of informal housing has also coincided with more dense informal settlements.

According to Ramunenyiwa (2020), municipal by-laws aim to regulate the development of human settlements in order to promote effective spatial planning and integrated service provision that factor the risks posed on human health, property and the environment. In addition, the *Prevention of Illegal Eviction from and Unlawful Occupation of Land Act 1998* seeks to de-escalate the frequency of informal settlements. Regrettably, the growth of informal settlements negatively points to the non-adherence of these by-laws. This is supported by empirical evidence presented by Mabaso (2019:117) who found that communities in the Umlazi Township disregarded spatial planning considerations by illegally settling on vacant lands. This practice was also established by UN Habitat (2015 in Sinharoy et al. 2019), which described informal settlements as residential settlements that have been constructed on land that is occupied illegally.

### The risk of floods

The negative characterisation of townships is indicative of the vulnerabilities that have rendered informal settlements in these geographies susceptible to floods. According to Hendricks and Van Zandt (2021), the susceptibility to hazards in townships is a consequence of the various social, physical and economic conditions that define the social and physical vulnerabilities of these geographies. Empirical studies have highlighted the consequences of the mentioned factors in causing disasters. For instance, a study by Mawasha and Britz (2021) using GIS methodologies ascertained that the vulnerability to flooding in the Alexandra Township is because of the proximity of housing (mostly informal) to the floodplains of the Juskei River. Napier and Rubin (2002) elucidated that people in Alexandra Township construct their houses on steep slopes that are susceptible to floods and landslides. Moreover, Napier and Rubin (2002:5) posited that the ‘… rising damp, poor indoor air quality and collapsing structures’ exposes these settlements to the disaster risks. In the aforementioned case studies, flood vulnerability arises from the nature of informal settlements themselves.

## Research setting: The Nelson Mandela Bay municipality

The Nelson Mandela Bay Municipality – a Category A municipality – is the largest municipality in the Eastern Cape covering a surface area of 1959 square kilometres (NMBM 2023b). The total population is estimated at 1 272 991 that is divided into 369 663 households (NMBM 2023a). Approximately 93.8% of the population live in formal housing, 6.2% live in informal housing while 37% of formal housing is attributed to Reconstruction and Development Programme (RDP) and other forms of government subsidised housing (NMBM 2023b).

The municipality faces several socioeconomic issues that are disproportionately distributed within the townships of Ibhayi, Northern Areas, Kariega and Motherwell. Among these is the unemployment rate that currently sits at 34.4% and the reliance on grants for 27.2% of households in the municipality (NMBM 2023a). Another prominent issue is the prevalence of crime, which mainly affects poor communities in townships. The proliferation of crime is shown in the murder rate, which sits at 71 per 100 000 – higher than the national average of 33.5 per 100 000.

Despite these negative population characteristics, the municipality is appropriately regarded as the Manufacturing hub of the Eastern Cape Province. Manufacturing contributes largely to the gross value added (GVA) (a measure of the production output) of the province and country respectively. In 2021, the sector accounted for 54.8% of the total GVA in the province and 4.11% nationally. This is despite a steady decline of 0.08% between 2016 and 2021 (NMBM 2023a). The stronghold of manufacturing has been aided by the development of economic infrastructure such as the Coega Industrial Development Zone, deep sea port of Ngqurha and the Port of Port Elizabeth, which have fostered the growth of industry and large scale built environments thereof (NMBM 2022). This has not only maintained the strength of the municipality’s manufacturing industries, but it has also attracted a large influx of skilled and unskilled migrants and immigrants who recolate to the municipality in search of employment and urban economic opportunities.

### Urbanisation in the municipality

The social dynamics of urbanisation have influenced the establishment and mushrooming of informal settlements concentrated in the townships. Considering the gentrification of the housing market in the municipality, poor migrants are excluded from participation (Mzileni 2021). This has perpetuated inaccessibility to formal housing for migrants and illegal immigrants who cannot afford formal accommodation. According to Williams-Bruinders and De Wit (2020), most migrants in the municipality survive on one dollar a day and therefore have to make use of alternative housing in the form of informal dwellings on vacant plots usually on the edge of cities and towns around the municipality. This practice contributes towards the ‘… uncontrolled growth of informal settlements flowing from unlawful land invasions’ (NMBM 2023b:201). Thus informal settlements have risen steadily in the municipality from about 76 in 2017 to 156 in 2023 (NMBM 2023b).

Urban informality is however a global phenomenon. The United Nations (2014) in Abunyewah et al. (2017) estimates that nearly one billion people live in informal settlements around the world. With projections estimating that the global population of people residing in cities will increase to 66% by 2050 (United Nations 2014), the number of informal settlements in urban geographies such as townships in the NMBM are likely to increase drastically. The effects of urban migration are derived according to the existing socioeconomic context of geographies. In the NMBM, these effects continue to follow the format of developing societies, whereby the influx of migrants and immigrants limits public service capacity and creates imbalances in the delivery of key social and economic development areas. These imbalances are clear from the enormous service delivery capacity challenges demonstrated by backlogs in the provision of housing, water, sanitation, refuse removal, tarring road and stormwater drainage as well as managing ageing infrastructure – all of which disproportionally affect townships and informal settlements in the municipality (NMBM 2023a).

The socioeconomic context of the municipality highlights that townships and informal settlements, in particular, face vulnerability in various forms. Much of this can be attributed to the legacies of Apartheid that saw townships inherit dilapidating infrastructure and weakened social and economic conditions (Wasserman et al. 2022). Progressively, studies have also accentuated that political factors in the form of disparity in the quality of services delivered in townships (particularly in informal settlements) and those delivered in residents formerly reserved for white people also widen the social vulnerability in townships. As a largely migrant population surviving on less than one US Dollar a day, informal settlement dwellers in the municipality rely significantly on the services of government to provide vulnerability-reducing goods and services. Therefore the failures of government capacity is sure to negatively impact their coping capacities in an environment with clear and evolving vulnerabilities.

## Results

### Flood risk

In the last 20 years, the municipality has faced invariable climatic conditions that have led to occasional heavy rainfall caused by thunderstorms and coastal weather systems (NMBM 2023b). The frequency of invariable climatic conditions has coincided with the increase in floods (NMBM 2023b) signifying that floods in the municipality are largely initiated by heavy rainfall. Historical disaster indexes of the municipality indicate that the municipality has experienced an increase in floods over the last four decades (NMBM 2023a). In addition, the disaster risk assessment of the municipality ranked floods as the highest disaster risk (SRK Consultants 2010). Drawing on the IVA, the following sections look into the vulnerability factors that advance heavy rainfall into flood risk within informal settlements in the townships.

#### Exposure

The exposure to the risk of floods in townships is a result of the location of human settlements. According to the NMBM (2023b), the municipality is grappling with informal housing being constructed on ‘stressed areas’ that are at risk of flooding during heavy rainfall. This study defines stressed areas as areas with a pronounced susceptibility to flooding. In the municipality, these areas include ‘… flood plains, drainage channels, catchments [*and*] storm water routes’ (NMBM 2023b:209). The Soweto-on-Sea and Veerplaas informal settlements are some common examples of urban informalities constructed on stressed areas – on the basis that these settlements are ‘… partly developed on the floodplain of the Chatty River’ (De Wit in Williams-Bruinders & De Wit 2020:128). This is aligned with the findings in De Risi et al. (2013), which demonstrated that the location of informal settlements rendered residents vulnerable to floods. Similarly, a study by Mwalimba et al. (2024) specified that the exposure to floods in urban informal settlements is very high because of the proximity of settlements to catchments. These studies confirm that flood vulnerability is a function of exposure, which is exacerbated by the tendency of informal settlements to be located in low-lying areas and river courses.

In line with this literature, a Missionvale informal settlement was found to be located on the edge of the Missionvale Saltpan Lake (between 5 and 60 metres above sea level) (Siyonqwana et al. 2015). The locality of this settlement is significant from a topographical and geomorphologic perspective. Topographically, the location experiences a regular flow of water coming from the inflow of salt water that is pumped out regularly and from the overland inflow from the surrounding areas. The location’s geomorphological composition is dominated by low-lying clay soils, which allow the pan to retain water for extended periods, thereby waterlogging the soil and preventing it from providing drainage (Siyonqwana et al. 2015). As a result, during heavy rainfall, the inflow of water can infiltrate low-lying houses because it will be voluminous and accelerated in low-lying areas. This argument is supported by studies which confirm that many informal settlements in South Africa are constructed on sensitive and fragile environments (such as flood plains, poorly drained land, wetlands, saltpans and steep slopes) that are unsuitable for safe housing and susceptible to floods (Morolle et al. 2022; Williams et al. 2019). In these studies and in this study, urbanisation is argued for the settlement on unsuitable land.

## Discussion

### Social factors driving exposure

Interestingly, the exposure to flood hazards is knowingly accepted by people in several informal settlements in the municipality’s townships. For instance, Siyonqwana et al. (2015) found that residents of a Missionvale Informal Settlement are well aware of the risk to floods but prefer the location because of its proximity to employment opportunities, health and educational facilities. This is also the case in an informal settlement in the KwaZakhele township, as found by Mngomezulu (2020). Evidently, people tend to prioritise the socioeconomic advantages presented by their location even when they have adequate knowledge of the danger presented by their occupation of that location. This finding is consistent with Huchzermeyer et al. (2013) and Satterthwaite et al. (2020) who cited that the opportunity to be close to employment opportunities, business activities and education institution is enough for people to live under the risk of floods.

Intriguingly in the present study, the ‘voluntarily hazard exposure’ of informal settlement dwellers amid the risk of floods is also influenced by a ‘sense of place’ or social attachment to location. According to DeMoss (2012:170), some former residents of an informal settlement in the Walmer Township (resettled in Wells Estate by urban authorities) ‘… felt that they have lost a sense of control compared to what they had in their former shacks’. This finding speaks to a social attachment to a location that would explain the reversion to old settlement plots after people have been resettled to safer sites (Lupindo 2015). Interestingly, this place attachment exist despite continued incursions of financial and psychosocial costs caused by floods in the studied areas. This voluntary exposure to flood hazard can also be explained by the fear of losing property by informal settlement dwellers. For example, Van Zyl (2023) and Dangazele (2022) found, in separate instances, that in an informal settlement in the Wells Estate located near the Markman Canal and prone to flooding, residents opted to subject themselves to the risk of floods by opposing their relocation to safer sites by local authorities. For these residents, the fear rested on losing their belonging, homes and plots once they leave for safer sites. On the other hand, in the Missionvale Township, people shared that it was not easy to move from the area because they felt that area is home and aligns with their cultural values (Siyonqwana, Heijne & Tele 2015).

These findings present an interesting prospect in which a ‘sense of place’ (whether derived from sentimentality or social and economic locational advantage) subjugates informal settlement residents to flood vulnerability. Napier and Rubin (2002) also offered a compelling argument by explaining that residing in these locations is sometimes a strategy employed by informal settlement dwellers to access government housing benefits quicker. This claim is, however, not supported in the evidence of this study. However, the notion of ‘sense of place’ remains a pertinent factor of hazard exposure in the literature as it is in the present study. For instance, covering the 2000 floods in the Alexandra Township, Batha (2000) uncovered that people refused to move from the banks of the Juskei River despite floods destroying their property. Their refusal, consistently with the present study’s findings, was because they feared losing their belongings and plots. Reflecting on the Alexandra Township floods, Napier and Rubin (2002) claimed that many of these people returned to the banks of the Juskei River to rebuild their homes after the water levels subsided. In the case where people did not return, they were replaced by other families that moved in.

### Critical infrastructure

Engineering perspectives highlight the inefficiencies of the built environment to withstand against the impacts of heavy rain in the municipality’s townships. Reference to the built environment is made on the structural integrity of human settlements and the critical municipal infrastructure meant to hold and network heavy rain during and after storms.

#### Housing infrastructure

With regard to the state of housing infrastructure, township informal settlements utilise informal means to build their houses in the NMBM (Sizani 2021). Consequently, their structures are often substandard and vulnerable to heavy rain and the current of run-off. This is aligned with the findings in Mwalimba et al. (2024), which attributed the vulnerability of urban informal settlements in Malawi to low-quality building materials in these geographies. In their study, however, Mwalimba et al. (2024) deduced that people have the resources and access to standard housing materials to build strong houses; however, they lack knowledge of building codes and standards. In contrast, the present study determined that the quality of informal settlements is based on economic status of the mostly migrant population that cannot afford to invest in hazard mitigating structures. This finding aligns with Abunyewah et al. (2017:240) who infer that the economic position of the mostly migrant population of informal settlement dweller ‘… inhibit [*them*] from investing in structural mitigation measures to reduce hazard impacts’.

Beyond the urban informality of organised and sporadic informal settlements, townships are also the hub of informal housing that is used as back-room accommodation for rental and extension of living space in serviced township plots (DeMoss-Norman 2015). According to Mzileni (2021:166), back room accommodation in the municipality’s townships became prominent from the 1970s primarily ‘… because many manufacturing sites in Port Elizabeth are located on abandoned lands situated near townships’. As a result, township property owners invested in turning unused yard space into backyard rooms to offer work-seeking migrants affordable accommodation (Mzileni 2021). Given that many people cannot afford standard building materials, people rely on informal means to build these structures (Morange 2002). According to DeMoss-Norman (2015), structures are constructed using building materials such as corrugated iron, wood, mud and plastics that are not strong enough to withstand the impact of excessive overland water. As such, inhabitants are exposed to the risk of flooding from the impact of overland flow of water (Tele 2018) and infiltration of underground water that damages their valuables. This is the case of Sakhasonke in the Walmer Township.

#### Stormwater reticulation system

In terms of the drainage of stormwater, the findings stress that stormwater infrastructure is insufficient in terms of quantity and size, and the existing infrastructure is dilapidated and not fully functional. Urban informal settlements are generally framed according to their infrastructural deficit and inadequacies (see King & Amponsah 2012; Le Quéré et al. 2020; Satterthwaite et al. 2020). According to Reis (2021), the inadequacy and deficiency of infrastructure in informal settlements ‘… is evident by both the lack of their initial development and their limited holding capacity’. This argument is apt for informal settlements in the NMBM for two reasons. Firstly, the municipality faces an infrastructural deficit of stormwater infrastructure in townships that is meant to redirect rainwater into catchments and tributaries that flow into municipal dams (Dangazele 2022). Secondly, the limited stormwater drains in the townships are ineffective (Zuze 2018). These findings indicate that the risk of floods in the townships is a result of the under-capacitation of existing stormwater reticulation systems against the rate of precipitation during heavy rainfall.

**The impact of waste management on stormwater infrastructure:** The challenge of functional stormwater reticulation systems in townships is propelled by communal negligence and a lack of waste management service delivery. Waste disposal practices by township communities along with the prevalence of pollution block drains affect their functionality (NMBM 2023b). For instance, Chirume and Issac (2023) found which township residents dump their solid waste upstream that gets swept into storm water drains by surges of rain. Consequently, the blocked stormwater drains prevent rain water from passing through storm water reticulation system into the network of tributaries and river catchments (Swanepoel et al. 2020).

The practice of illegal waste disposal is well informed in the literature suggesting that it is not unique to informal settlements in the townships of the NMBM. For instance, Pelling (2003) established that informal settlement dwellers tend to use the available storm drains as refuse areas. This finding was also established in early empirical evidence by McGranahan et al. (2001) in their assessment of social vulnerability in urban spaces. While the context of their study was not on urban informal settlements, their findings serve to highlight that people inhabiting ‘… poor urban areas are often exposed to harmful perturbations in and around their houses which are created primarily due to the lack of adequate public services’ (Nsorfon 2014:30). In this regard, the lack of adequate public services is uncollected solid waste.

It is important to notice that, while informal settlement dwellers share the responsibility of polluting with neighbouring formal settlement counterparts, *unserviced* informal settlements are the main instigators of illegal dumping (Matebese 2019). In both cases, informal settlement dwellers expectedly face the worst effects of the blocked drains from the resultant floods. The consequences of illegal waste disposal are not isolated to the practice of illegal dumping itself. Rather, they exist in combination with the lack of coordinated waste management by the municipality. This finding supports Abunyewah et al. (2017) in their argument that urban authorities tend to side line informal settlements from their planning interventions. In its IDP, the NMBM acknowledged this shortcoming citing that there are informal settlements that are not provided with waste disposal and collection facilities and service (NMBM 2023b). Moreover, the establishment of new informal settlements creates a backlog of areas that are not included in the municipality’s planning and budgeting. As such, planning interventions in informal settlements is limited. This finding is supported in Abunyewah et al. (2017:239), which found that informal settlements ‘… are located outside the planning schemes of urban areas’. This presents governance issues that challenge government in provisioning waste management services to these areas. As a result, their coping capacity to floods is reduced.

In South Africa, waste management is the prerogative of municipalities who must ensure that they adopt regular refuse disposal and cleaning of streets and storm water infrastructure (Republic of South Africa 2000). Competence in these areas can be argued to both directly and indirectly minimise the risk of floods as it would contribute towards the reduction of blocked storm water and manage the flow of heavy rainfall. Regrettably, Swanepoel et al. (2020) found that the municipality does not clear debris that is accumulated in storm water drains in townships as frequently as it should. Furthermore, the municipality does not employ efficient and proper waste management to reduce the waste in townships. For instance, only ‘… 67% of known informal settlements receive integrated waste handling services’ (NMBM 2023a:9). As a result, debris from the human waste and vegetation is washed into stormwater drains and blocks rain water from entering the stormwater system. Funding constraints pose a serious issue in the municipality realising the service provision of stormwater drainage maintenance. DeMoss (2012) states that this is because informal settlement residents do not pay taxes for municipal services such as refuse pickup and sewage among others. Evidently, flood vulnerability in informal settlements is also a result of political marginalisation (Sutherland 2019).

## Conclusion

The study has revealed that township informal settlements in the NMBM are on a continual rise owing to the rural-urban migratory patterns observed in the municipality. The growth of informal settlements coincides with the development of dense settlements that progressively become hotspots to flood disasters in the municipality. The study has highlighted that the effects of flood vulnerability are multifaceted as it is not limited to the exposure of hazards in these settlements. Rather, there are interactions between socioeconomic factors that directly and indirectly influence the presence of vulnerability in both its biophysical and social form. Much of this has to do with the socioeconomic marginalisation of informal settlement dwellers, who by virtue of being low-skilled migrants, have to adopt ‘survival’ practices and behaviours that subject them to flood vulnerability. In addition, these people experience a political marginalisation in the delivery of important infrastructural service delivery that can reduce the exposure to biophysical hazards and build their coping capacities. In accordance with the IVA framework, this characterisation determines the makeshift nature of their housing structure, selection of location, energy choices and planning interventions (or lack thereof) by local authorities.
